# Adult adrenal ganglioneuroblastoma with an extensive metastasis of the skull: case report and review of the literature

**DOI:** 10.3389/fonc.2026.1787041

**Published:** 2026-07-30

**Authors:** Willem Vandenhoudt, Tony Van Havenbergh, Frederic Van Havenbergh

**Affiliations:** 1 Department of Neurosurgery, University Hospitals Leuven, Leuven, Belgium; 2 Department of Neurosurgery, ZAS Hospitals Antwerp, Antwerp, Belgium

**Keywords:** ganglioneuroblastoma, metastasis, neurosurgery, oncology, case report

## Abstract

**Background:**

Ganglioneuroblastoma (GNB) is a malignant neoplasm of the autonomic nervous system on the spectrum of peripheral neuroblastic tumors (PNT), exceedingly rare in adults with only a limited number of cases documented in literature.

**Case presentation:**

We present the case of a 57-year-old male who was found to have a large nodular lesion in the left adrenal gland, which was later confirmed as a ganglioneuroblastoma following adrenalectomy, with multiple skeletal metastases and, most notably, a large, growing and destructive metastasis in the right frontoparietal region of the skull with extradural extension and invasion of the superior sagittal sinus. We discuss the patient’s clinical, radiological, and pathological findings in correlation with the current literature and recommendations regarding this type of tumor.

**Conclusion:**

This case, associated with a review of the literature, highlights the malignant potential of ganglioneuroblastoma in adults and emphasizes the need for early diagnosis, a multidisciplinary treatment approach, and further investigation into optimal management strategies for this rare entity.

## Introduction

Ganglioneuroblastoma (GNB) is a malignant neoplasm of the autonomic nervous system on the spectrum of peripheral neuroblastic tumors (PNT) (1). It originates from primitive neuroectodermal cells of the neural crest that migrate during embryonic life, giving rise to the sympathetic ganglia, and the adrenal medulla (2). GNB usually is predominantly a pediatric malignancy and is extremely rare in adults with fewer than 50 cases reported in literature to date. The most frequent site is the adrenal gland, other sites are the retroperitoneum, the brain, and the mediastinum (3, 4).

Beyond its rarity, adult GNB poses unique diagnostic and therapeutic challenges because adults frequently present with atypical symptoms. This often results in delayed diagnosis and advanced-stage disease at presentation. Furthermore, the biological behavior of adult GNB appears to differ from that of pediatric GNB with a greater propensity for metastatic disease and poorer overall outcomes. Because prospective studies in adults are lacking, current management strategies are largely extrapolated from pediatric protocols including surgery, chemotherapy, radiotherapy and meta-iodobenzylguanidine (MIBG) therapy. Consequently, each well-documented adult case provides valuable information regarding diagnostic and therapeutic challenges unique to adult patients.

We describe the case of a 57-year-old male with an incidental 6 cm adrenal mass with an extensive metastatic lesion in the right frontoparietal region of the skull (9 cm diameter extracranially, 8 cm diameter intracranially) with extradural extension and invasion of the superior sagittal sinus. This case illustrates the diagnostic challenges associated with an atypical clinical presentation, the limitations of currently available treatment strategies for adult disease, and the importance of individualized multidisciplinary management.

## Case presentation

**Figure 1:**
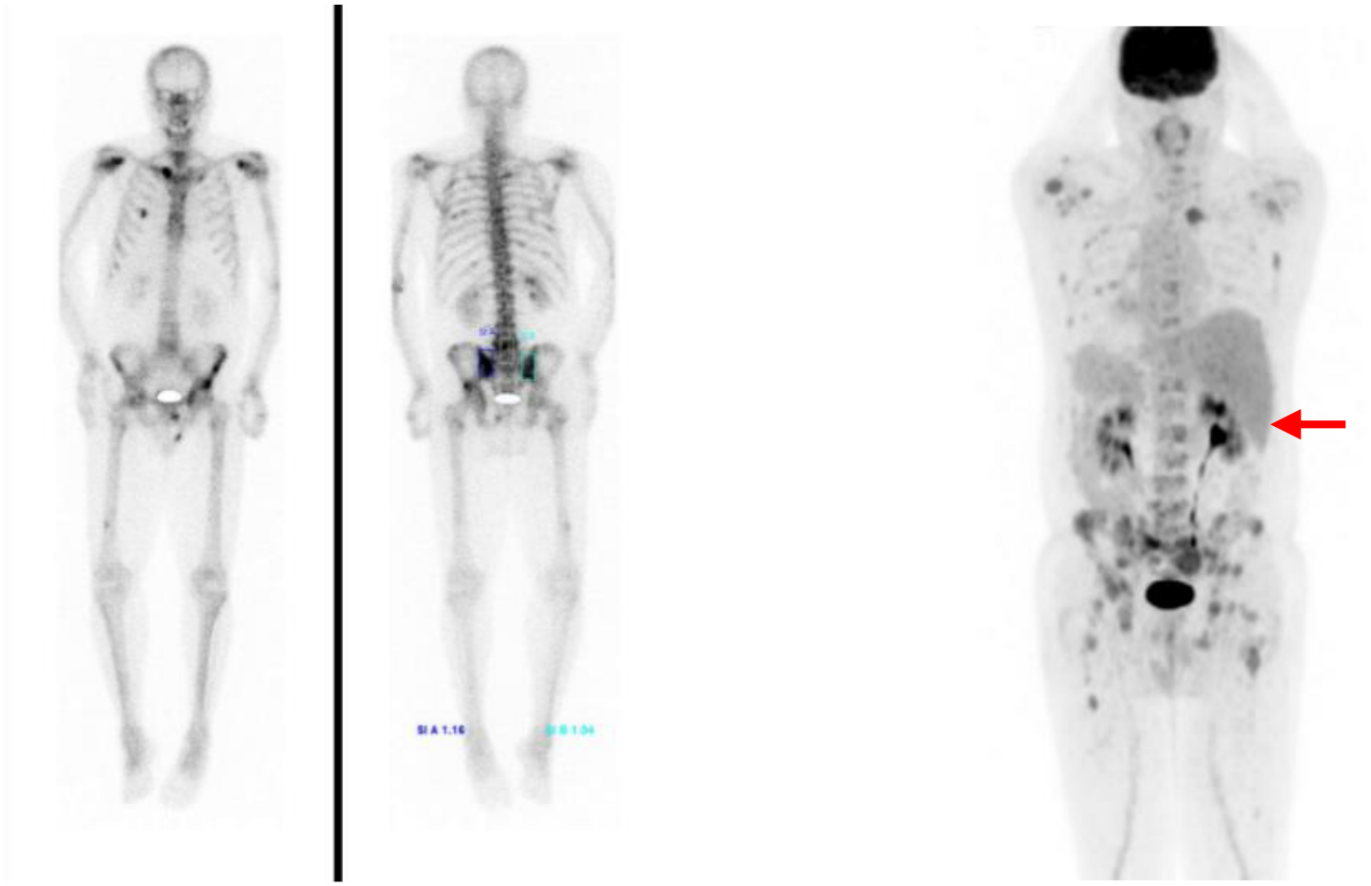
SPECT- and ^18^F-FDG PET-CT, respectively, showing multiple osteolytic bone lesions at the thoracolumbar spine, pelvis and rib cage, with also a large nodular lesion at the level of the left adrenal gland (red arrow).

We report on the case of a 57-year-old patient who presented with an 8-week history of progressive right-sided sciatica based on an atypical lesion at the level of the L5 endplate with compression of the S1 nerve root on the right. Magnetic resonance imaging of the lumbar spine demonstrated an atypical lesion involving the L5 vertebral endplate with compression of the right S1 nerve root. SPECT- and ^18^F-FDG PET-CT revealed the presence of multiple osteolytic bone lesions at the thoracolumbar spine, pelvis, and rib cage, strongly suspicious for malignant tumor tissue, with also a large nodular lesion at the level of the left adrenal gland (**Figure 1**). A CT-guided biopsy of the left iliac crest did not yield a diagnosis. A micro-exploration at L5-S1 on the right was performed, with the goal of both decompressing the right S1 nerve root and obtaining a tissue diagnosis (undetermined).

Such non-diagnostic outcomes may occur in neuroblastic tumors due to factors such as tumor heterogeneity and sampling error. There was a strong suspicion of a primary tumor in the left adrenal region, for which an exploratory laparoscopy was proposed at an early stage but was initially refused by the patient.

A few months later, the patient eventually underwent a laparoscopic left adrenalectomy. Microscopically the tumor showed a ganglioneuroma component on one side and a neuroblastoma component on the other, consistent with a diagnosis of nodular ganglioneuroblastoma (cT2NxM1, NGS negative). Following multidisciplinary discussion, systemic chemotherapy was recommended but declined by the patient. The patient subsequently received MIBG therapy and radiotherapy, resulting in initial disease control. Treatment was complicated by hematological toxicity necessitating regular blood transfusions. Neuron-specific enolase (NSE) was used as a biomarker to monitor treatment response.

**Figure 2:**
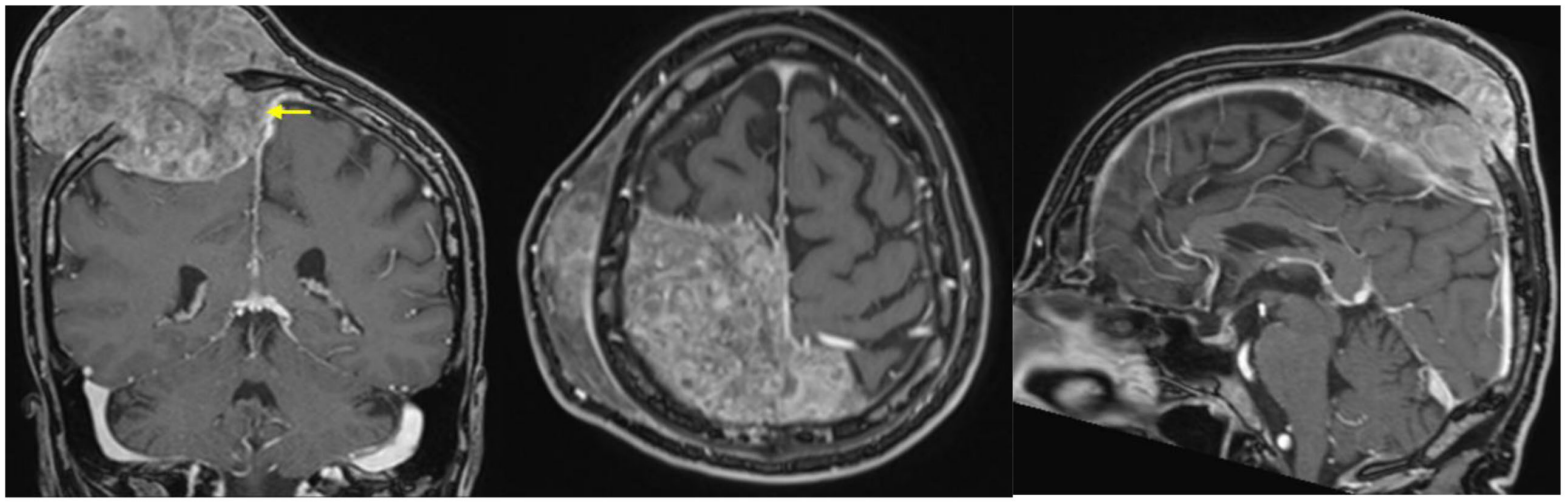
Pre-operative contrast-enhanced T1-weighted magnetic resonance imaging (MRI) of the brain (left: coronal plane, middle: axial plane, right: sagittal plane) shows a contrast-enhancing lesion emerging from the right frontoparietal calvarium with extradural extension. Invasion of the superior sagittal sinus can be seen (yellow arrow).

However, more than one year after initial diagnosis patient presented with a growing mass in the right frontoparietal region of the skull. CT and MRI of the brain revealed an exceptionally large, well-demarcated and destructive, most likely metastatic skull lesion with extradural extension and invasion of the superior sagittal sinus (**Figure 2**). Preoperative assessment of the superior sagittal sinus was based on the contrast-enhanced MRI. Additional vascular imaging with CT angiography or MR venography was not performed because it was not considered necessary for surgical decision-making. This represents a limitation of the present report, as the degree of sinus patency could not be assessed in detail.

After surgery for a right-sided pathologic hip fracture, a neurosurgical procedure was performed to reduce tumor load and minimize both the extracranial and intracranial mass effect.

**Figure 3:**
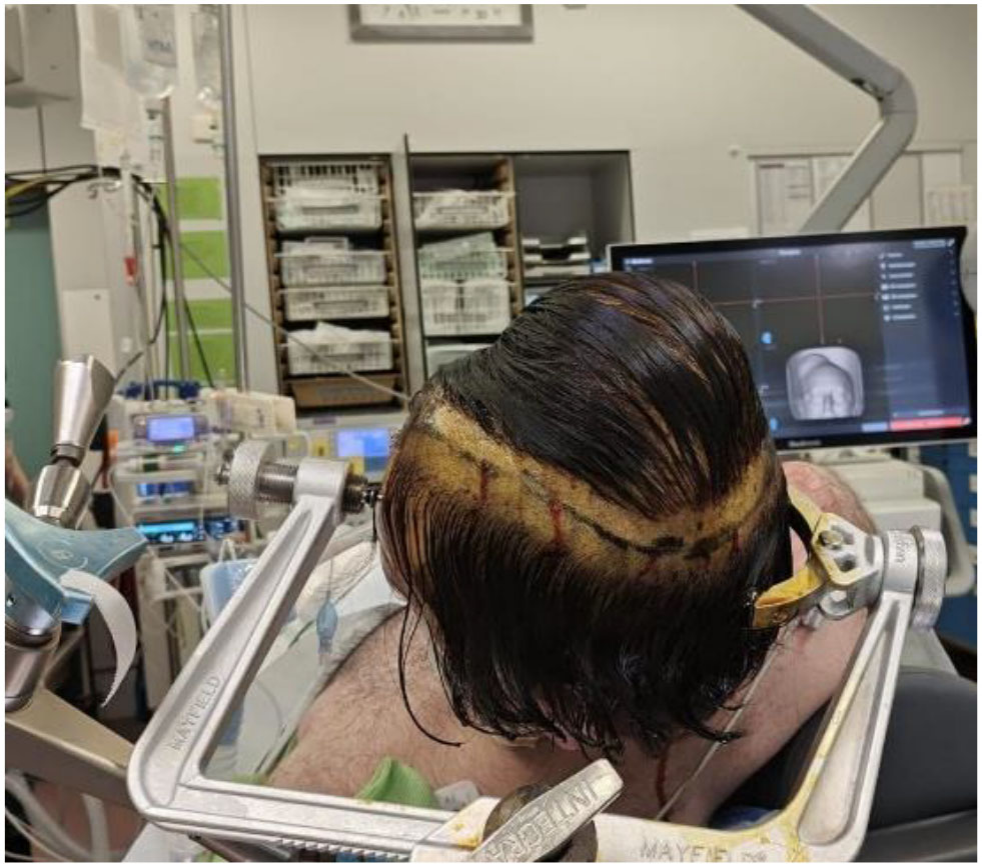
Patient positioning in dorsal decubitus with a cushion under the right shoulder and the head fixed in the Mayfield clamp and turned slightly to the left.

**Figure 4:**
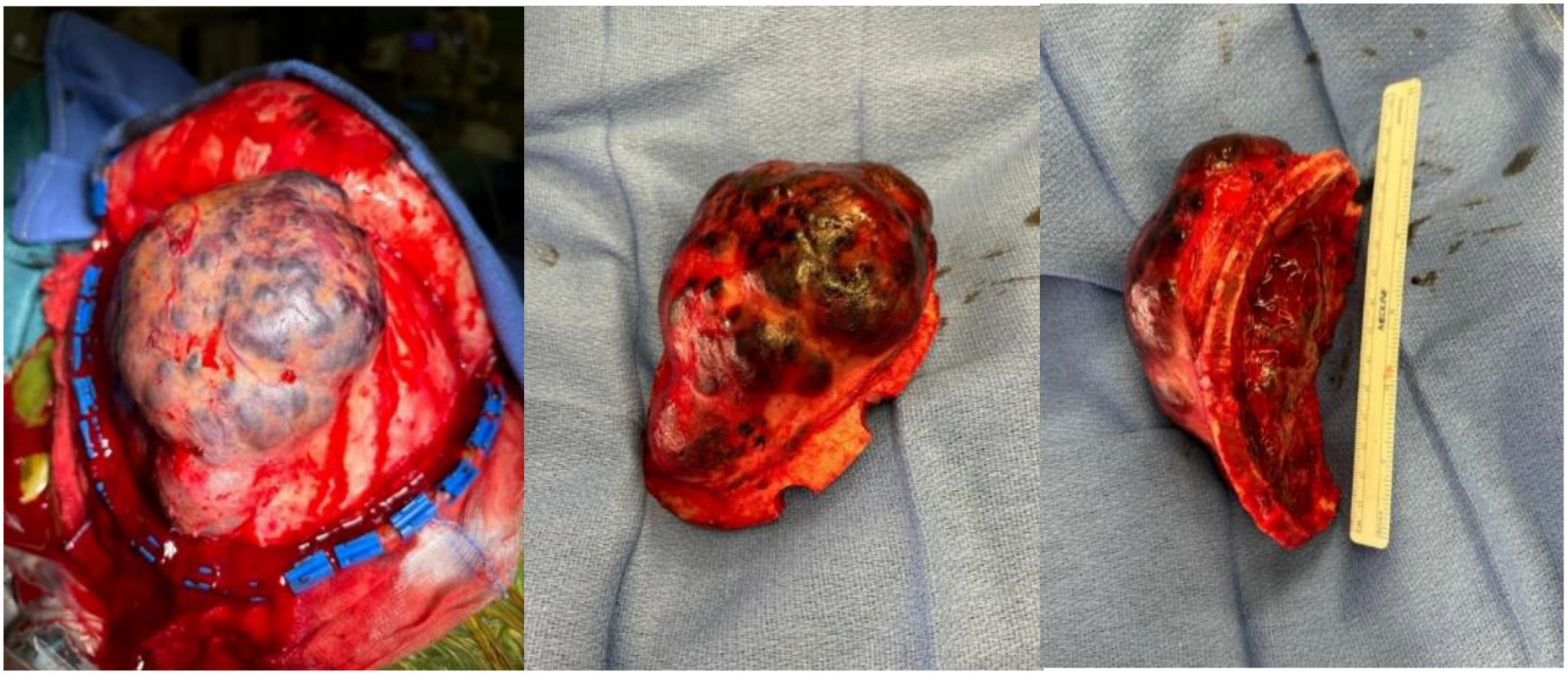
Tumoral exposure using a C-shaped skin incision, angled towards the temporal area, around the lesion. A near-total removal of the tumor was achieved.

The patient was positioned in dorsal decubitus with a cushion under the right shoulder and the head fixed in the Mayfield clamp and turned slightly to the left, as shown in **Figure 3**. A resection was performed using a C-shaped skin incision, angled towards the temporal area, around the lesion. Following exposure, the tumor was found to be firm in consistency, bluish in appearance, and highly vascularized, with a large extracranial subcutaneous and intraosseous component extending into an intracranial extradural component. A near-complete resection of the lesion, measuring approximately 9 cm in diameter, was achieved (**Figure 4**), leaving a small part of tumor at the level of the superior sagittal sinus. Afterwards a cranioplasty with bone cement was performed to repair the calvarial deficit. The total duration of the procedure, including general anesthesia, was approximately 5 hours. Perioperatively there was a repeated need for transfusion due to the extensive bleeding of the lesion.

Histopathological examination of the resected tumor showed a fibrous, partially necrotic, multinodular lesion with predominantly neuroblastomatous cells and several atypical ganglion cells interspersed confirming the diagnosis of metastasis from the known nodular ganglioneuroblastoma. Immunohistochemical studies were positive for chromogranin, synaptophysin, and partially positive for S100. Ki-67 staining showed a tumor cell proliferation index of approximately 20%. There was no aberrant p53 expression. Sox10 staining was negative in the neuroblastoma cells.

**Figure 5:**
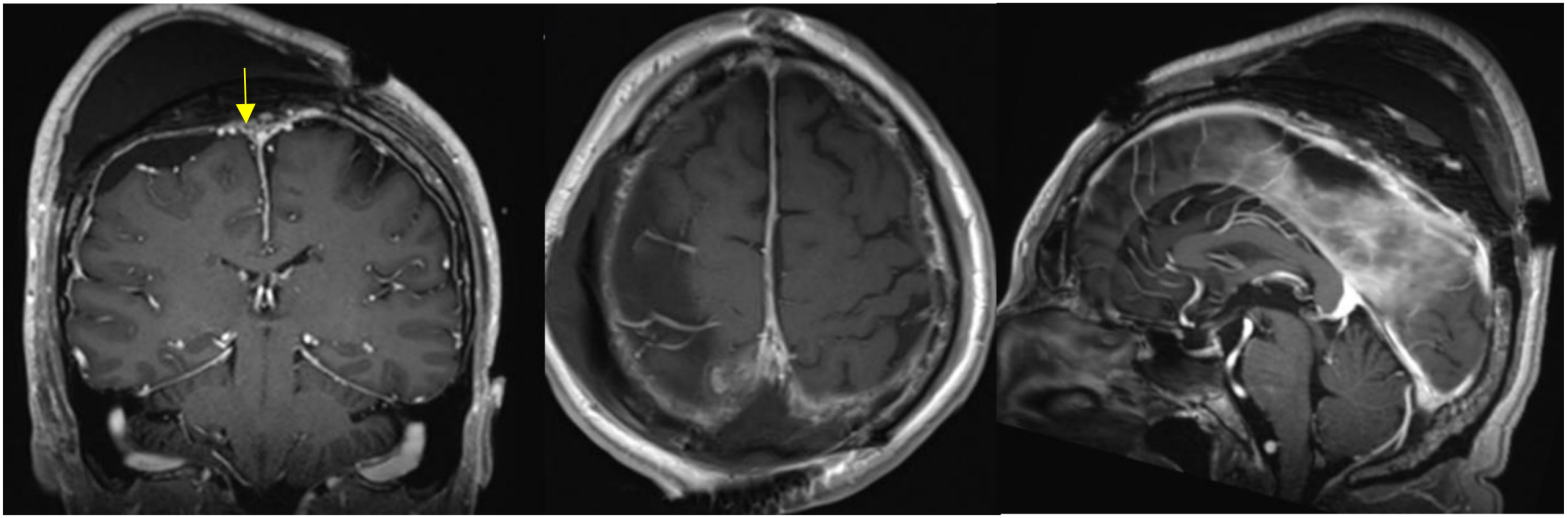
Postoperative contrast-enhanced T1-weighted magnetic resonance imaging (MRI) of the brain (left: coronal plane, middle: axial plane, right: sagittal plane) shows a near-maximal resection with only a limited remnant adjacent to the superior sagittal sinus (yellow arrows).

A postoperative MRI was reassuring, showing no signs of ischemia with only a limited tumor remnant adjacent to the superior sagittal sinus (**Figure 5**). The patient was discharged from the hospital a few days after surgery.

Adjuvant chemotherapy was initially deferred because of persistent transfusion dependence resulting from bone marrow failure caused by extensive metastatic bone marrow infiltration. Despite supportive treatment, the disease continued to progress with the development of extensive osseous and hepatic metastases. Following multidisciplinary discussion, the anticipated benefits of further oncological treatment were considered to be outweighed by the patient’s deteriorating clinical condition, and a decision was made to transition to best supportive care. The patient died of progressive disease approximately 6 months later.

## Discussion

Ganglioneuroblastoma (GNB) in adults remains an exceptionally rare entity, representing less than 1% of all neuroblastic tumors, which predominantly occur in childhood (1, 5, 6). The disease arises from neural crest cells capable of differentiating into both ganglionic and neuroblastic components, leading to a spectrum of biological behaviors ranging from indolent to highly aggressive forms (2, 4, 7, 8). Published data about adrenal neuroblastic neoplasms are presented in detail in **Table 1**.

**Table 1:** Overview of adrenal neuroblastic neoplasms previously reported in literature.

Reference (year)	Age / sex	Primary site	Treatment	Pathology	Metastasis / recurrence	Recommendation
Deslarzes et al. (2022) (3)	74 / F	Left adrenal gland	Complete adrenalectomy; no adjuvant therapy	Nodular GNB	NA	Primarily surgical resection +/- selective adjuvant therapy in advanced disease
Mizuno et al. (2010) (7)	53 / M	Right adrenal gland	Complete adrenalectomy followed by treatment for osseous recurrence (systemic therapy and palliative management)	Nodular GNB	Lumbar spine (2 years after surgery)	Primarily surgical resection and close follow-up (+/- selective adjuvant therapy in advanced disease)
Jrebi et al. (2014) (10)	22 / F	Right adrenal gland	Surgical excision as primary therapy; selected patients received adjuvant chemotherapy and/or radiotherapy	GNB	NA	Surgical excision as primary therapy; adjuvant chemotherapy and/or radiotherapy in selected patients
Jrebi et al. (2014) (10)	51 / F	Adrenal gland	Lost to follow-up	NB	NA	Surgical excision as primary therapy; adjuvant chemotherapy and/or radiotherapy in selected patients
Vassalo et al. (2021) (14)	22 / M	Right adrenal gland	Laparoscopic adrenalectomy without adjuvant chemotherapy or radiotherapy	Intermixed GNB	NA	CT and MRI to further characterize masses and useful in pretreatment risk stratification based on clinical criteria and image-defined risk factors
Zhang et al. (2019) (16)	75 / F	Left adrenal gland	Radical adrenalectomy; no standardized adjuvant regimen recommended because of rarity	NB	Lung and brain (22 months after surgery)	Surgical resection alone +/- local radiotherapy, chemotherapy considered in disseminated disease
Heidari et al. (2018) (18)	38 / M	Right adrenal gland	Open adrenalectomy with complete tumor excision; no adjuvant therapy	Nodular GNB	NA	Primarily surgical resection and close follow-up (clinical, imaging including CT and MIBG scintigraphy)
Ding et al. (2019) (19)	27 / F	Left adrenal gland	Complete surgical excision including adrenalectomy; postoperative surveillance without immediate adjuvant therapy	GNB	NA	Combination therapy (surgery +/- radio- and/or chemotherapy) and active follow-up
Joana et al. (2010) (20)	65 / F	Right adrenal gland	Laparoscopic adrenalectomy; no adjuvant therapy	GN	NA	Surgical resection + close clinical and imaging follow-up

M, male; F, female; GNB, ganglioneuroblastoma; NB, neuroblastoma; GN, ganglioneuroma; NA, not applicable.

Epidemiological studies demonstrate that adult-onset neuroblastic tumors often present at an advanced stage, with poorer survival compared to pediatric cases (2, 5, 6). Franks et al. and Jrebi et al. both emphasized the atypical and often delayed presentation in adults, noting that the disease course may initially appear indolent but ultimately confers poor prognosis due to resistance to conventional pediatric treatment regimens (9, 10). In our patient, the progression from adrenal primary tumor to widespread bone metastases and a massive calvarial lesion reflects this aggressive biological potential.

Histopathologically, adult GNB tends to exhibit more mature components compared with pediatric neuroblastoma yet paradoxically shows a worse clinical outcome (8). This apparent discrepancy may relate to inherent differences in tumor biology, including lower MYCN amplification rates but higher frequency of unfavorable tumor kinetics (11). Moreover, as Whittle et al. highlighted, the lack of adult-specific treatment protocols forces clinicians to extrapolate from pediatric experience, which may not adequately address the pharmacodynamic and tolerance differences observed in older patients (12).

Bone metastases, such as those observed in our case, are a well-recognized feature of advanced neuroblastic tumors and correlate with worse outcomes (4, 13, 14). However, central nervous system and skull metastases are exceptionally rare, reported in less than 5% of adult cases (15, 16). The extensive frontoparietal skull metastasis in our patient underscores the importance of comprehensive neuroimaging at diagnosis and during follow-up.

Therapeutically, surgical resection remains the cornerstone for localized disease, whereas multimodal treatment including radiotherapy, systemic chemotherapy, and MIBG therapy is recommended for disseminated cases (12, 17, 18). In the present case, despite an initially good response to surgery, MIBG, and radiotherapy, disease progression continued, illustrating the limitations of current therapeutic strategies. Similar observations were made by Jrebi et al. and Zhang et al., who reported variable responses to multimodal therapy and overall limited survival in adult patients despite aggressive management (10, 16). This suggests that current treatment regimens may be insufficient for aggressive adult GNB (4, 19).

The role of surgery in metastatic GNB is largely palliative, focusing on mass reduction and symptom control. In our patient, neurosurgical intervention was essential to reduce both intracranial pressure and cosmetic deformity, while minimizing neurological complications. As previously reported by Rogowitz et al. and Franks et al., multidisciplinary management integrating endocrinology, oncology, radiotherapy, and neurosurgery is crucial for optimizing outcomes in these complex presentations (6, 9).

Overall, adult GNB remains a diagnostic and therapeutic challenge. The current case adds to growing evidence that adult-onset disease exhibits distinct clinical behavior, often with aggressive metastatic spread and limited responsiveness to pediatric treatment protocols. Future studies should aim to define the molecular and immunohistochemical characteristics of adult GNB, which may inform targeted therapeutic strategies and improve prognosis. Until such data are available, early recognition, complete surgical resection when feasible, and individualized multimodal therapy remain the cornerstones of management.

## Conclusion

Ganglioneuroblastoma in adults is an exceptionally rare disease with unpredictable biological behavior and generally poor prognosis compared to pediatric cases. This case illustrates the malignant potential of adrenal GNB and its capacity for widespread metastasis. The literature consistently indicates that adult-onset neuroblastic tumors often present at an advanced stage and respond less favorably to conventional pediatric treatment regimens. While surgical resection remains the mainstay for localized disease, optimal management of disseminated or recurrent GNB requires a tailored, multimodal approach involving surgery, radiotherapy, systemic chemotherapy, and, when indicated, MIBG therapy. Despite these efforts, survival outcomes remain limited, underscoring the need for adult-specific treatment protocols and improved molecular characterization to guide targeted therapy. Ultimately, early recognition, comprehensive imaging, and multidisciplinary collaboration are essential to achieve the best possible outcomes. Continued documentation of adult cases, such as the present report, is vital to deepen understanding of disease behavior and to support the development of evidence-based therapeutic strategies for this rare and challenging malignancy.

## Data Availability

The original contributions presented in the study are included in the article/supplementary material. Further inquiries can be directed to the corresponding author.
